# Yinchen lipid-lowering tea attenuates lipid deposition in a fatty liver model by regulating mitochondrial dysfunction through activation of AdipoR1/AMPK/SIRT1 signaling

**DOI:** 10.1007/s13205-024-04204-2

**Published:** 2025-01-13

**Authors:** Xilin Luo, Yuanyuan Fang, Wei Wang, Meiling Tong, Bin Qin, Jinyu Cao, Yinjie Yang

**Affiliations:** Department of Preventive Treatment of Disease Centre, Nanchong Chinese Medicine Hospital (Nanchong Traditional Chinese Medicine Hospital Affiliated to North Sichuan Medical College), 200 Jingyuling Zhengjie Road, Shunqing District, Nanchong City, Sichuan Province 637000 People’s Republic of China

**Keywords:** Non-alcoholic fatty liver disease, Yinchen lipid-lowering tea, Lipid deposition, Mitochondrial dysfunction, AdipoR1/AMPK/SIRT1 signaling pathway

## Abstract

This study investigated the ameliorative effects of Yinchen lipid-lowering tea (YCLLT) on Non-alcoholic fatty liver disease (NAFLD), the specific mechanism involved was also studied. We modeled hepatocellular steatosis with HepG2 cells and intervened with different concentrations of YCLLT-containing serum. Lipid deposition was assessed by oil red O staining and AdipoR1 expression was analyzed by Western blot. The hepatocyte steatosis model was further treated with YCLLT-containing serum and/or silencing AdipoR1. Lipid deposition was observed by oil red O staining. Flow cytometry was used to detect apoptosis and mitochondrial membrane potential. The levels of TNF-α, IL-6, MDA, 8-OHdG, and ATP were analyzed by ELISA or the corresponding kits. The mitochondrial structure was observed by transmission electron microscopy. The expression of AdipoR1/AMPK/SIRT1 signaling pathway factors was analyzed by Western blot, and co-localization of SIRT1 and immunofluorescence. The results revealed that YCLLT attenuated lipid deposition, inhibited the levels of inflammatory factors TNF-α and IL-6, reduced the levels of MDA and 8-OHdG, up-regulated the ATP content and mitochondrial membrane potential, and promoted the expression of AdipoR1, p-LKB1, p-AMPKα, SIRT1, and PGC-1a in a cellular model of NAFLD. Further, silencing of AdipoR1 inhibited the ameliorative effect of YCLLT in the NAFLD cell model. Altogether, Yinchen lipid-lowering tea attenuates lipid deposition in a fatty liver model by improving mitochondrial function via activating AdipoR1/AMPK/ SIRT1 signaling.

## Introduction

Non-alcoholic fatty liver disease (NAFLD) is a clinical syndrome characterized by fatty vacuolar degeneration and excessive accumulation of fat in hepatocytes without a history of heavy alcohol consumption, which manifests itself in varying degrees of hepatic lesions ranging from lipid deposition to severe inflammatory responses in hepatitis, liver fibrosis, and even cirrhosis (Pouwels et al. 2022; Guo et al. 2022). It is closely related to obesity, diabetes, insulin resistance, and genetic susceptibility, and is also one of the main causative factors leading to hepatocellular carcinoma, and its incidence is increasing year by year (Lee and Lui 2022). Up to now, there is no effective treatment for NAFLD, lifestyle interventions (diet, weight management, physical exercise, etc.) are still the main therapeutic strategies, and drugs such as metformin, pioglitazone, and vitamin E have a certain effect on NAFLD, but their safety and side effects need to be further evaluated, e.g., corticosteroids may increase the risk of infections and metabolic disorders (Paternostro and Trauner 2022) so there is a need to find new therapeutic drugs for NAFLD.

Yinchen lipid-lowering tea (YCLLT) is derived from the Yinchenhao decoction (classical formulae, documented in the Treatise on Febrile Diseases and the Synopsis of Prescriptions of the Golden Chamber) of Zhang ZhongJing, a medical saint, and modern network pharmacology proves that Yinchenhao decoction can regulate blood lipids by inhibiting lipogenesis and metabolism, promoting cholesterol excretion, antioxidant, regulating inflammation, and vascular remodeling (Chen et al. 2021; Chen et al. 2015). Experimental studies have shown that Yinchen can reduce hepatic triglyceride (TG) deposition and delay disease progression in high-fat-induced NAFLD mice (Cai et al. 2018, 2019). The preliminary study showed that YCLLT was clinically effective in regulating the levels of total cholesterol (TC), TG, and low-density lipoprotein cholesterol (LDL-C) in patients with hyperlipidemia (Liu et al. 2022). Furthermore, YCLLT, as a traditional Chinese medicine prescription, has ingredients derived from natural herbs, which have fewer side effects and are safer in use, which has certain clinical advantages in its application (Chen et al. 2021). However, the ameliorative effect of YCLLT on NAFLD is unclear.

Studies have shown that activation of AMP protein kinase (AMPK) leads to decreased lipogenesis and increased fatty acid oxidation and lipolysis in adipocytes in vitro and in vivo (Long et al. 2019; Zhou et al. 2022). In addition, AMPK further leads to deacetylation of downstream sirtuin1 (SIRT1) targets and regulation of lipid deposition by regulating SIRT1 activity (Zhang et al. 2023b). In the liver, AdipoR1 induces AMPK activation (Li et al. 2022). Furthermore, the liver is a central organ for lipid metabolism, and lipid deposition is affected by lipid metabolism, whereas lipid metabolism and energy metabolism are related to mitochondrial function, which is regulated by SIRT1 through deacetylation of PGC-1a (Zhang et al. 2023a). Mitochondrial dysfunction is characterized by abnormal mitochondrial morphology (swelling, cristae structural disorders), reduced ATP production, and a decrease in the number of functional mitochondria. Mitochondrial dysfunction triggers apoptosis of hepatocytes, exacerbation of hepatic inflammation, and promotes the onset and progression of hepatic fibrosis (Zhao et al. 2023). Therefore, whether YCLLT regulates mitochondrial dysfunction through AdipoR1/ AMPK/SIRT1 signaling to attenuate hepatocyte inflammation and lipid deposition in high-fat models deserves further investigation.

In this study, we used HepG2 cells to establish a model of hepatocellular steatosis, and the effects of YCLLT on lipid deposition, inflammatory injury, mitochondrial dysfunction, and the AdipoR1/AMPK/SIRT1 signaling pathway of the model of hepatocellular steatosis, and to explore the possible molecular mechanisms by which YCLLT improves the hepatocellular steatosis, so that it will provide experimental bases for prevention and control of NAFLD as well as for the development of effective drugs.

## Material and methods

### Preparation of YCLLT medicated serum (YCLLT-MS)

Five SPF-grade SD rats were provided by Chuanbei Medical College (SCXK (Chuan) 2023–0018). YCLLT consisted of 1:1:1:1:1 of Yinchen (*Artemisia capillaris* Thunb.): raw shanzha (*Crataegus pinnatifida* Bunge): Jiaogulan (*Gynostemma pentaphyllum* Thunb.): Juemingzi (*Cassia obtusifolia* L.): Lotus leaves. Rats were given YCLLT by gavage (1 ml/100 g) once/day for 2 weeks, and blood samples were collected immediately after rat execution and serum was extracted. All rats were approved by the Experimental Animal Ethics Committee of West China Hospital (20,230,113,006).

### Preparation of free fatty acids (FFAs)

31.7 μL of oleic acid (OA, Sigma-Aldrich, USA) was mixed well with 968.3 μL PBS to obtain 100 mM of OA mother liquor. 25.6 mg of palmitic acid (PA, Sigma-Aldrich, USA) was dissolved in 1000 μL of isopropanol and completely dissolved and filtered using 0.22 μm microporous filter membrane to obtain 100 mM PA mother liquor. A 3 mM mixture of FFAs (2:1 ratio of OA to PA) was prepared as stock solution.

### Cell model of lipid accumulation

HepG2 cells were purchased from the Chinese Academy of Sciences. Cells were cultured in Dulbecco's modified Eagle's medium (DMEM, Gibco, CA) supplemented with 10% fetal bovine serum (FBS, Gibco, CA), 100 units/mL penicillin, and 100 μg/mL streptomycin at 37 °C 5% CO_2_ conditions. Logarithmic growth phase HepG2 cells were taken and incubated with 1 mM FFAs to stimulate lipid accumulation for 24 h.

### Cellular interventions

HepG2 cells were divided into control group, model group, 10% YCLLT-MS group, 15% YCLLT-MS group, 20% YCLLT-MS group, YCLLT-MS + AdipoR1 siRNA group, and YCLLT-MS + AdipoR1 NC group. Control cells were cultured normally using culture medium for 24 h. Cells in the model group were cultured with 1 mM FFAs for 24 h. Group with different concentrations of YCLLT-MS was cultured for 24 h with 1 mM FFAs and different concentrations of YCLLT-MS. YCLLT-MS + AdipoR1 siRNA group: HepG2 cells were transfected with AdipoR1 siRNA (RiBoBio, Guangzhou), and after transfection, the transfected HepG2 cells were incubated with 1 mM FFAs and YCLLT-MS for 24 h. YCLLT-MS + AdipoR1 NC group: HepG2 cells were transfected with AdipoR1 NC (RiBoBio, Guangzhou), and after transfection, post-transfected HepG2 cells were cultured with 1 mM FFAs and YCLLT-MS for 24 h.

### Oil Red O staining

Cultured cells of each group were washed twice with PBS, then fixed with 4% paraformaldehyde for 20 min, rinsed with 60% isopropanol (Sigma-Aldrich, USA) for 20–30 s. Mayer hematoxylin staining solution (Sigma-Aldrich, USA) was added for 1–2 min, and oil red O buffer (Sigma-Aldrich, USA) was added for 1 min, Distilled water was added to cover the cells, which were observed and photographed under a microscope (DMI1, LEICA, Germany), and the software Imagine PRO was used to analyze the oil red O results.

### Measurement of TNF-α, IL-6, MDA, ATP, and 8-OHdG levels

The levels of tumor necrosis factor-α (TNF-α), interleukin-6 (IL-6), malondialdehyde (MDA), adenosine triphosphate (ATP), and 8-hydroxy-2-deoxyguanosine (8-OHdG) were detected by using the kit (ZCiBio, Shanghai) following the kit instructions.

### Flow cytometric detection of apoptosis

The precipitates of each group of cells were obtained, and the cells were resuspended with 500 μL of Binding Buffer, then 5 μL of Annexin V (KeyGen Biotech, Nanjing) was added and gently blown, and then 5 μL of PI was added and mixed well, and the reaction was carried out at room temperature and protected from light for 15 min, and then detected and analyzed by FC500 flow cytometer (Beckman Coulter, USA).

### Mitochondrial membrane potential (ΔΨmit)

The cells were taken from each group and centrifuged at 1500 r/min for 5 min to obtain the cell precipitate. The cells were resuspended with 500 μL of JC-1 working solution (Beyotime, Beijing), incubated in an incubator at 37 °C and 5% CO_2_ for 20 min, then centrifuged at 1500 r/min for 5 min, the supernatant was discarded, and the cells were washed twice with 1 × Incubation Buffer, each time with 1 mL, and the last 500 μL of 1 × Incubation Buffer was used to resuspend the cells, and then detected and analyzed by FC500 flow cytometer (Beckman Coulter, USA).

### Transmission electron microscopy (TEM)

Cells were pre-fixed with 3% glutaraldehyde (Sinopharm Chemical Reagent Co., Ltd., Shanghai), re-fixed with 1% osmium tetroxide, dehydrated step by step with acetone, embedded with Epon-812 pure embedding agent (EMCN, Beijing), and ultrathin sections of 60 ~ 90 nm were made with an ultrathin sectioning machine and dropped onto a copper mesh. The sections were stained with uranyl acetate (EMCN, Beijing) for 15 min, and then with lead citrate for 2 min. A JEM-1400FLASH transmission electron microscope (JEOL, Japan) was used for image acquisition.

### Quantitative RT-PCR (qRT-PCR) analysis

Trizol reagent (Invitrogen, Thermo Scientific, USA) was used to extract total RNA from cells. A reverse transcription reaction was used to synthesize cDNA. SYBR Green PCR Master Mix (Applied Biosystems, Foster City, CA), cDNA, and gene-specific primers were used for RT-PCR. Forward primer for Acox1: 5’-CACAAGTAAACCAGCGTGTAAA-3’, reverse primer 5’-GTTCTTAGCCCACTCAAACAAG-3’. Forward primer for PPARγ: 5’-AGATCATTTACACAATGCTGGC-3’, reverse primer: 5’-TAAAGTCACCAAAAGGCT TTCG-3’. Forward primer for Fasn: 5’-CCATCTACAACATCGACACCAG-3’, reverse primer: 5’-CTTCCACACTATGCTCAGGTAG-3’. Forward primer for Pdk4: 5’-CAAGATG CCTTTGAGTGTTCAA-3’, reverse primer: 5’-GGTCTTCTTTTCCCAAGACAAC-3’. Forward primer for GAPDH: 5’-TGACTTCAACAGCGACACCCA-3’, reverse primer: 5’-CACCCTGTTGCTGTAGCCAAA-3’. All primers were synthesized by Sangon Bio (Shanghai, China). The reaction was carried out at a temperature of 95 °C for 30 s, followed by a temperature of 95 °C for 10 s, then a temperature of 72 °C for 15 s, for a total of 40 cycles. The calculation of the relative expression of each target gene was performed using the 2^−ΔΔCT^ method.

### Western blot analysis

The experiment involved the inoculation of cells onto a plate. RIPA cell lysis buffer (Beyotime, Beijing) was added to induce cell lysis, followed by centrifugation to obtain the supernatant containing proteins. Then, for protein separation, SDS-PAGE gel electrophoresis was employed, transfer of the proteins onto a PVDF membrane. The membrane was subsequently sealed using 5% skimmed milk powder at room temperature. Following this, the primary antibodies (AdipoR1, 1:2000; LKB1, 1:2000; p-LKB1, 1:2000; AMPKα, 1:1000; p-AMPKα, 1:2000; SIRT1, 1:1000; PGC1α, 1:1000; β-actin, 1:50,000; all antibodies procured from Wuhan Abclonal) were incubated on the membrane. The membrane was washed thrice with PBS before the addition of the secondary antibody for further incubation. After another round of PBS washing, a luminous solution was added, and the final results were observed through the utilization of the ECL chemiluminescence system.

### Immunofluorescence staining

The cell climbing slices were washed three times with PBS for 5 min each time, and membrane-breaking solution was added dropwise to cover the cells, and incubated for 10 min at room temperature, bovine serum was blocked at room temperature for 20 min, a mixture of SITR1 (Bioss, Beijing) and TOMM20 (Abcam, UK) or AdipoR1 (Abcam, UK) primary antibody was added dropwise and incubated overnight at 4 °C, washed three times with PBS for 5 min each time, and the corresponding secondary antibodies (FITC-labeled goat anti-rabbit, CY3-labeled goat anti- mouse, Servicebio, Wuhan), incubated at 37 °C for 30 min, and DAPI incubated at room temperature for 10 min. Images were captured using an A1 confocal microscope (Nikon, Japan) and the fluorescence intensity of all acquired images was measured using the Image-J analysis system (National Institutes of Health, USA).

### Statistical analysis

Results were presented as mean ± SEM. Differences were evaluated by a two-way analysis of variance (ANOVA) with Bonferroni post-hoc test among groups, and *P* value < 0.05 was considered as statistical significance. All regular plots were displayed using GraphPad Prism version 7.0 (La Jolla, CA, USA).

## Results

### Effect of YCLLT-MS on lipid deposition and AdipoR1 in hepatocytes of lipid accumulation model

**Fig.1:**
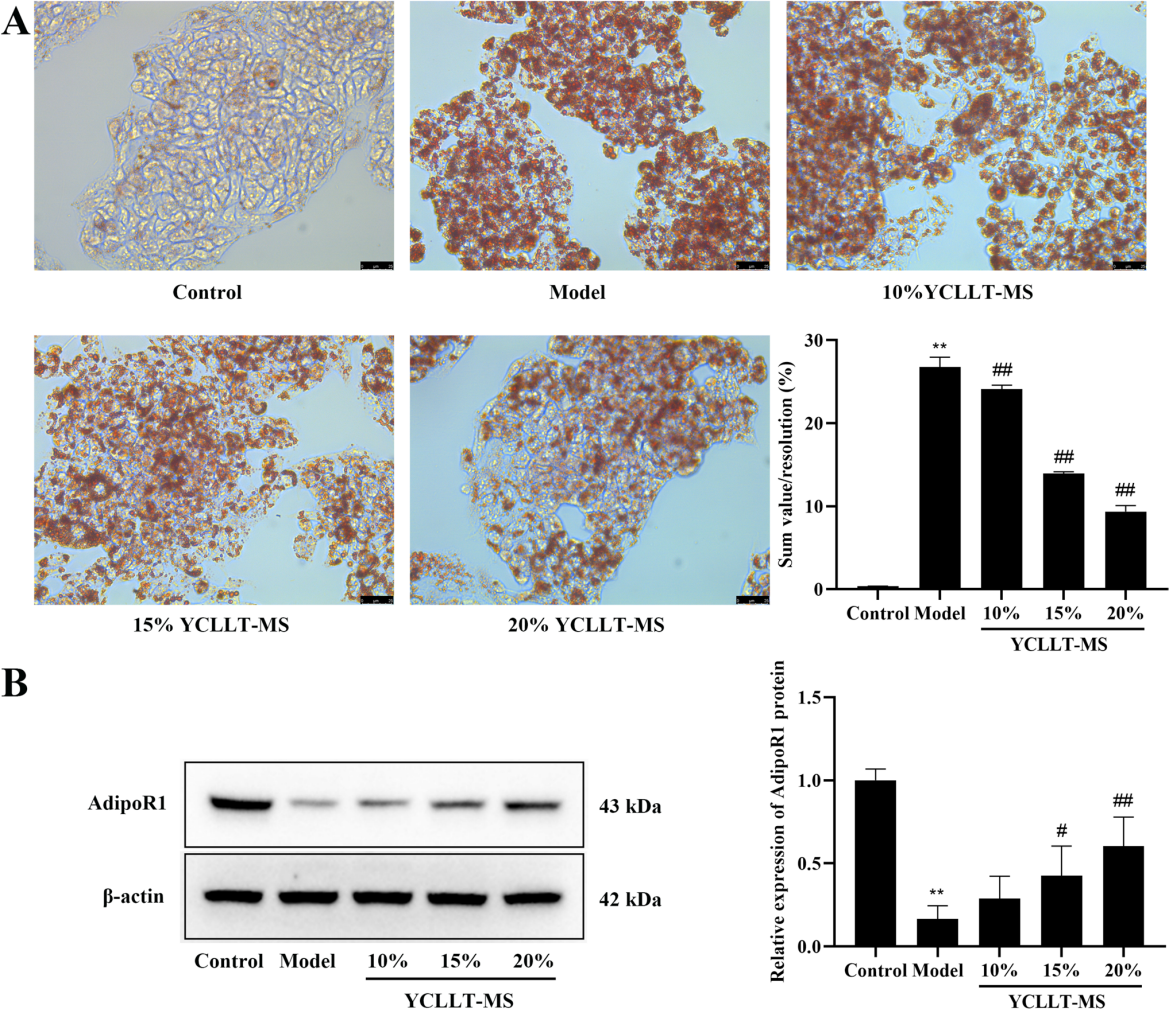
Effect of YCLLT-MS on lipid deposition and AdipoR1 in hepatocytes of lipid accumulation model. **A** Lipid deposition was observed with Oil Red O staining (Scale bar, 25 μm). **B** Expression levels of AdipoR1 in cells detected by Western blot. Data were represented as mean ± SEM. Compared with control group, **P < 0.01; compared with model group, #*P* < 0.05, ##*P* < 0.01

First, we observed the effect of YCLLT-MS on lipid deposition and AdipoR1 in hepatocytes of lipid accumulation model. As shown in Fig. 1. Lipid deposition was significantly increased and protein expression of AdipoR1 was significantly decreased in the model group compared with the control group (*P* < 0.01), however, 15% YCLLT-MS and 20% YCLLT-MS could significantly reduce the lipid deposition and significantly increase the protein expression of AdipoR1 of the model cells, and 20% YCLLT-MS had a more pronounced effect, thus, 20% YCLLT-MS was used for subsequent experiments.

### Effects of silencing AdipoR1 on lipid deposition, inflammatory injury, and lipid metabolism in the YCLLT-MS action hepatocyte lipid accumulation model

**Fig.2:**
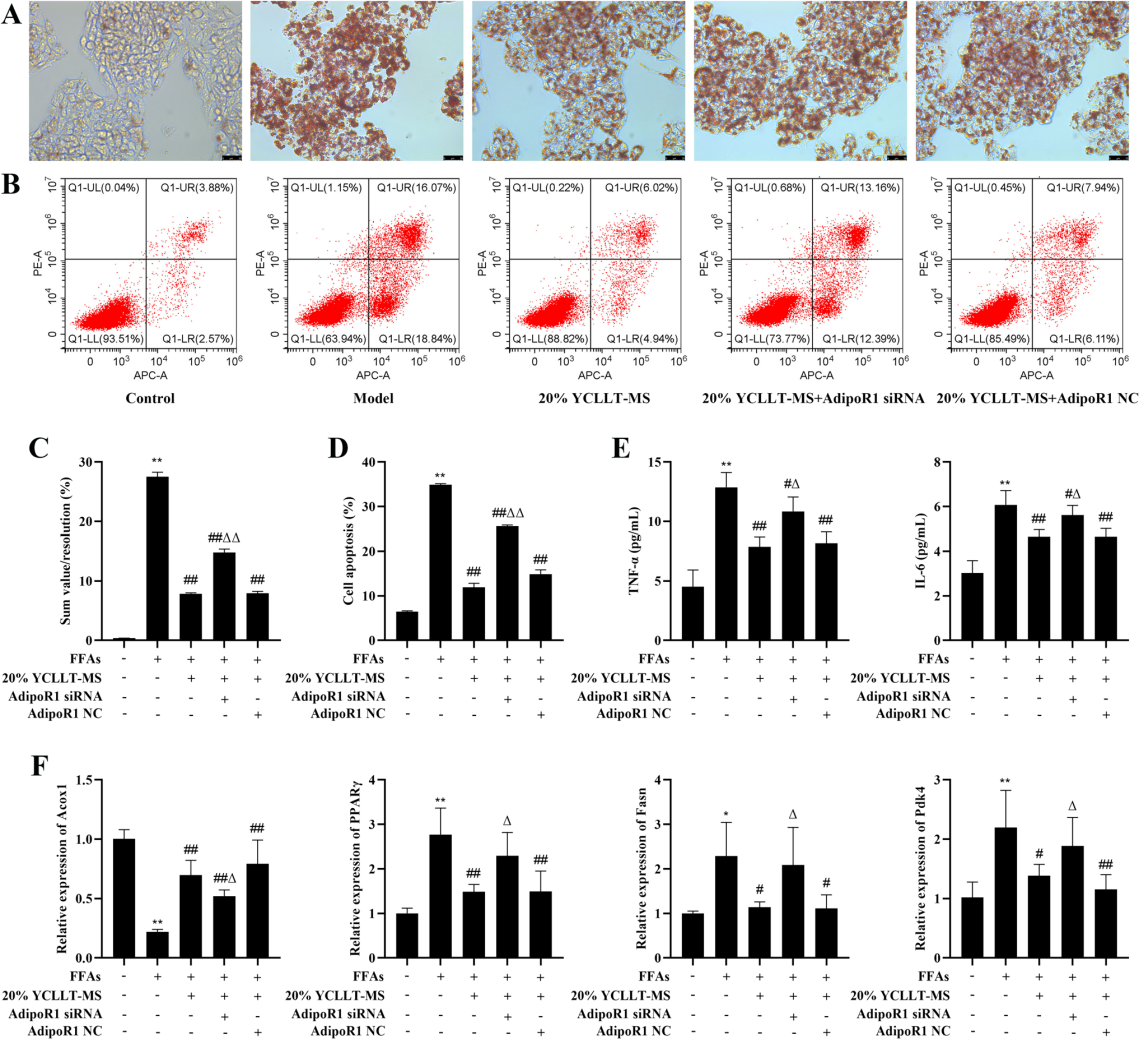
Effects of silencing AdipoR1 on lipid deposition, inflammatory injury, and lipid metabolism in the YCLLT-MS action hepatocyte lipid accumulation model. **A** Oil Red O staining to observe lipid deposition (Scale bar, 25 μm). **B** Flow cytometric analysis of representative images of apoptotic cells. **C** Quantitative analysis of oil red O staining. **D** Quantitative analysis of apoptosis. **E** The levels of TNF-α and IL-6 in each group of cells were detected with the kits. **F** Expression levels of Acox1, PPARγ, Fasn, and Pdk4 in cells detected by qRT-PCR. Data were represented as mean ± SEM. Compared with control group, *P < 0.05, **P < 0.01; compared with model group, #*P* < 0.05, ##*P* < 0.01; compared with 20% YCLLT-MS group, Δ*P* < 0.05, ΔΔ*P* < 0.01

Next, we examined the effect of silencing AdipoR1 on lipid deposition, inflammatory injury, and lipid metabolism in the YCLLT-MS action hepatocyte lipid accumulation model. We found that lipid deposition and apoptosis were significantly increased, and the levels of TNF-α, IL-6 and the expression of PPARγ, Fasn, and Pdk4 were also significantly enhanced, and the expression of Acox1 was significantly attenuated in the AdipoR1 siRNA + 20% YCLLT-MS group compared with the 20% YCLLT-MS group (*P* < 0.05, Fig. 2), indicating that YCLLT-MS may inhibit lipid deposition and inflammatory injury in the hepatocyte lipid accumulation model through AdipoR1.

### Effect of silencing AdipoR1 on mitochondrial dysfunction in the YCLLT-MS action hepatocyte lipid accumulation model

**Fig.3:**
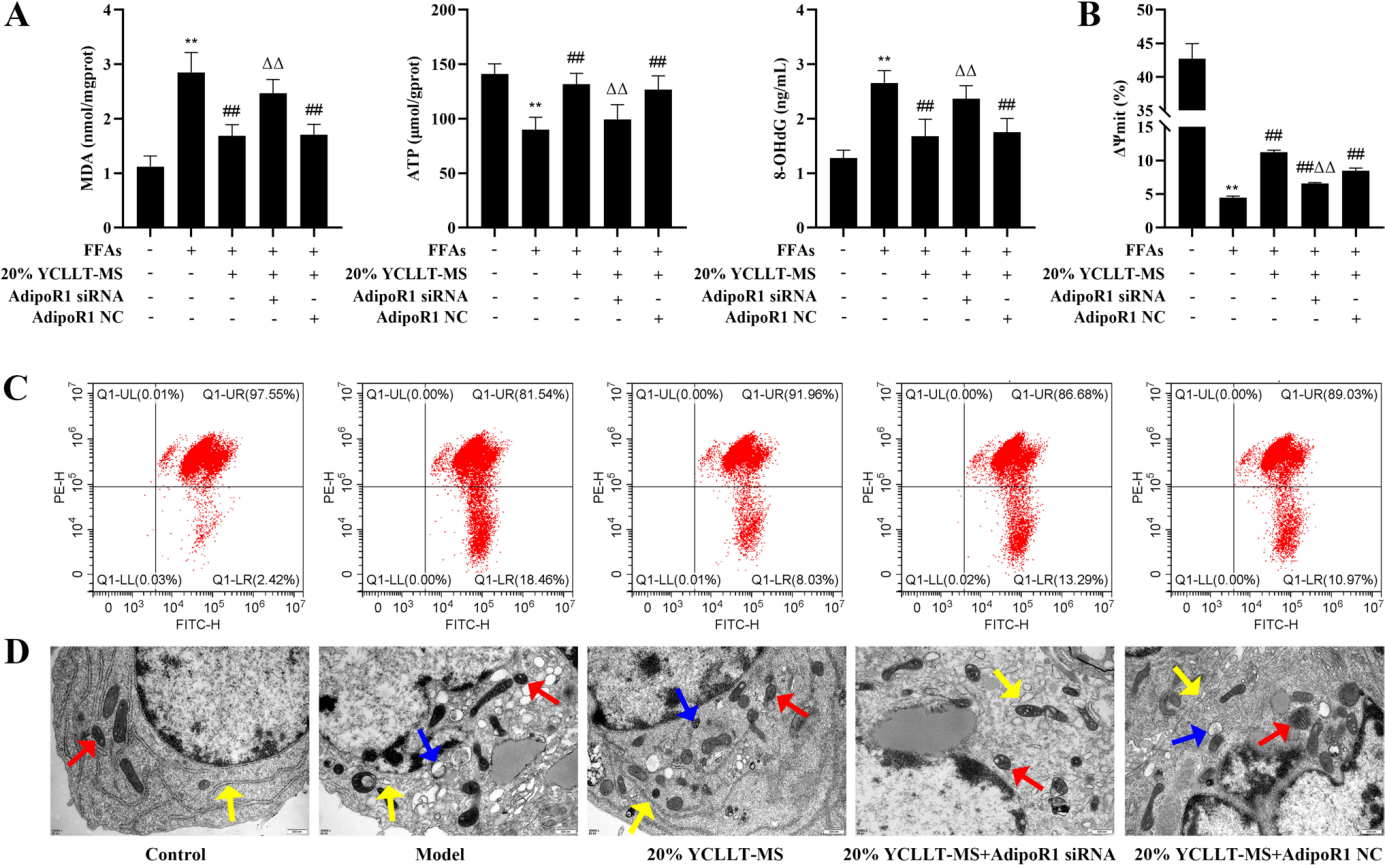
Effect of silencing AdipoR1 on mitochondrial dysfunction in the YCLLT-MS action hepatocyte lipid accumulation model. **A** The levels of MDA, ATP, and 8-OHdG in each group of cells were detected with the kits. **B**–**C** Flow cytometric analysis of mitochondrial membrane potentials. **D** Mitochondrial ultrastructure observed by transmission electron microscopy (× 20,000, Scale bar, 500 nm, Red arrows: normal or crumpled mitochondria, yellow arrows: normal or mildly expanded rough endoplasmic reticulum, blue arrows: autophagy). Data were represented as mean ± SEM. Compared with control group, **P < 0.01; compared with model group, ##*P* < 0.01; compared with 20% YCLLT-MS group, ΔΔ*P* < 0.01

In this part, we analyzed the effect of silencing AdipoR1 on mitochondrial dysfunction in the YCLLT-MS action hepatocyte lipid accumulation model. The results revealed that the levels of MDA and 8-OHdG were significantly increased, and ATP and mitochondrial membrane potential were remarkably decreased in the model group compared with the control group, however, 20% YCLLT-MS significantly improved the levels of the above indexes in the model (*P* < 0.01, Fig. 3A–C). Compared with the 20% YCLLT-MS group, the AdipoR1 siRNA + 20% YCLLT-MS group markedly elevated the levels of MDA and 8-OHdG, and significantly attenuated ATP and mitochondrial membrane potential (*P* < 0.01, Fig. 3A–C). TEM results showed that hepatocyte cytoplasmic mitochondria in the model group were significantly wrinkled (with significantly higher membrane density, darker color under electron microscopy), and some of the rough endoplasmic reticulum was mildly dilated, and lipid droplets and autophagy were seen in the cytoplasm. 20% YCLLT-MS ameliorated the hepatocyte injury described above, and hepatocyte injury was enhanced in the AdipoR1 siRNA + 20% YCLLT-MS group compared to the 20% YCLLT-MS group (Fig. 3D), suggesting that YCLLT-MS may ameliorate mitochondrial dysfunction through AdipoR1 in a hepatocyte lipid accumulation model.

### Effect of silencing AdipoR1 on the AdipoR1/AMPK/SIRT1 signaling pathway in the YCLLT-MS action hepatocyte lipid accumulation model

**Fig.4:**
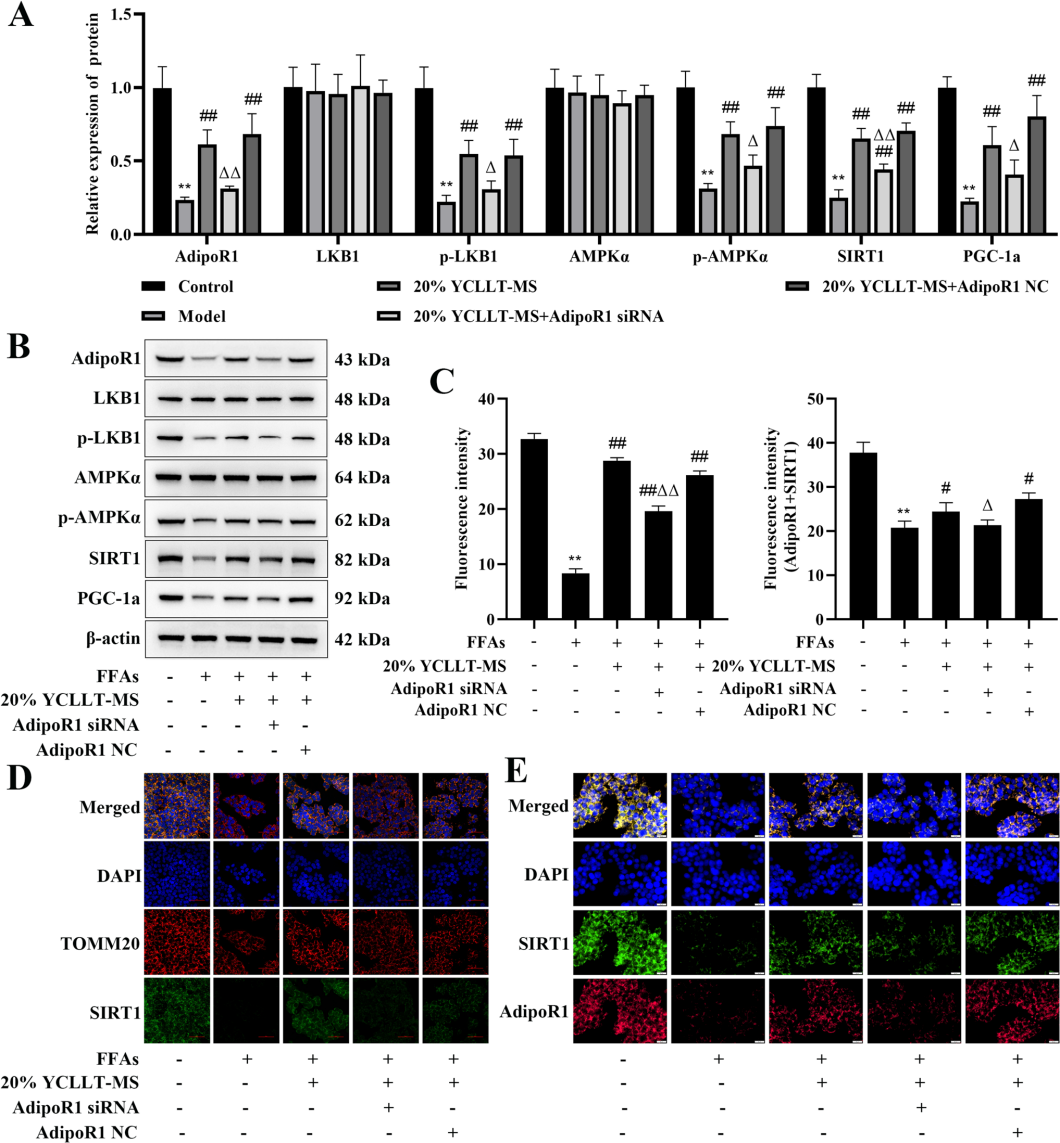
Effect of silencing AdipoR1 on the AdipoR1/AMPK/SIRT1 signaling pathway in the YCLLT-MS action hepatocyte lipid accumulation model. **A** Expression levels of AdipoR1, LKB1, p-LKB1, AMPKα, p-AMPKα, SIRT1, and PGC-1a in cells detected by Western blot. **B** Quantitative analysis of WB results in (**A**). **C** Fluorescence intensity analysis of immunofluorescence staining. **D** Immunofluorescence staining for TOMM20 and SITR1 (× 40, Scale bar, 20 μm). **E** Immunofluorescence staining for AdipoR1 and SITR1 (× 40, Scale bar, 20 μm). Data were represented as mean ± SEM. Compared with control group, **P < 0.01; compared with model group, #*P* < 0.05, ##*P* < 0.01; compared with 20% YCLLT-MS group, Δ*P* < 0.05, ΔΔ*P* < 0.01

Further, we analyzed the effects of silencing AdipoR1 on the AdipoR1/AMPK/SIRT1 signaling pathway in the YCLLT-MS action hepatocyte lipid accumulation model. The results showed that the protein expression of AdipoR1, p-LKB1, p-AMPKα, SIRT1, and PGC-1a was significantly reduced in the model group compared with the control group, and 20% YCLLT-MS reversed the protein expression of the above indicators; however, the protein expression of AdipoR1, p-LKB1, p-AMPKα, SIRT1, and PGC-1a was significantly decreased in the AdipoR1 siRNA + 20% YCLLT-MS group compared to the 20% YCLLT-MS group (*P* < 0.05, Fig. 4A, B). The fluorescence intensity of SITR1 (Fig. 4C, D) and AdipoR1 + SITR1 (Fig. 4C, E) of the cells was significantly reduced in the model group compared with the control group (*P* < 0.01). 20% YCLLT-MS intervention significantly enhanced the fluorescence intensity of SITR1 and AdipoR1 + SITR1 (*P* < 0.05). The fluorescence intensity of SITR1 and AdipoR1 + SITR1 was significantly decreased in the AdipoR1 siRNA + 20% YCLLT-MS group compared to the 20% YCLLT-MS group (*P* < 0.05), showing that YCLLT-MS promoted the AdipoR1/AMPK/SIRT1 signaling pathway in the hepatocyte lipid accumulation model.

## Discussion

In the present study, we used YCLLT-MS to treat the FFAs-induced NAFLD model in HepG2 cells. Here, we show that YCLLT-MS had a certain extent of ameliorating effect on lipid accumulation in the NAFLD cell model. Moreover, our data indicates that YCLLT-MS promoted the activation of the AdipoR1/AMPK/SIRT1 signaling pathway and may inhibit lipid deposition, inflammatory injury, and ameliorate mitochondrial dysfunction in the NAFLD cell model by regulating AdipoR1.

Lipid accumulation is caused by an imbalance between hepatic fat synthesis and catabolism, which is an important cause of NAFLD (Ipsen et al. 2018). In addition, evidence showed that inflammatory mediators were significantly elevated in FFAs-induced hepatocyte culture supernatants (Su et al. 2018). Our in vitro results also showed that FFAs induction significantly increased lipid deposition, the levels of IL-6 and TNF-α in HepG2 cells, and treatment by YCLLT-MS significantly reduced lipid deposition and inflammatory factor release.

PPARγ is typically expressed at low levels under normal physiological conditions. However, its expression gradually increases as hepatic lipid accumulation progresses, playing a key role in the development of NAFLD by regulating processes such as lipid synthesis and uptake (Chen et al. 2023; Qiu et al. 2023). Previous studies have found that PPARγ promotes NAFLD formation by stimulating downstream Fasn expression, and specific knockdown of PPARγ in hepatocytes resulted in a decrease in the expression of both lipid synthesis and uptake-related genes and a reduction in the degree of hepatocyte lipid accumulation (Liu et al. 2019; Xiao et al. 2023). Additionally, it has been shown that Acox1 is not only associated with the development of hepatic lipid accumulation, but the inactivation of hepatocyte Acox1 also leads to hepatic oxidative stress (Ding et al. 2022). In this study, the expression of PPARγ, Fasn, and Pdk4 was significantly increased and the expression of Acox1 was significantly reduced in FFAs-treated HepG2 cells, but the YCLLT-MS treatment reversed the expression levels of all of the above indexes, which showed that YCLLT-MS alleviated the processing burden in the state of lipid overload in hepatocytes and exerted a lipid-lowering effect, which was similar to the results of other reports (Park et al. 2019; Ji et al. 2019).

Excessive lipid accumulation causes oxidative stress and release of inflammatory factors, which in turn exacerbates regulatory disorders such as cellular dysfunction and mitochondrial disorders, leading to the development of more serious diseases such as NAFLD fibrosis (Ipsen et al. 2018; Chen et al. 2020). The level of ATP can be used as an evaluation indicator of mitochondrial status and activity (Choi et al. 2023). Decrease in intracellular ATP levels leads to a slowdown in oxidative phosphorylation, which results in disturbances in energy metabolism in hepatocytes (Görigk et al. 2022). The attenuation of mitochondrial membrane potential is one of the key indicators of mitochondrial dysfunction, and the stability of mitochondrial membrane potential is necessary to maintain normal cellular physiological processes, as well as to maintain the mitochondria for oxidative phosphorylation and ATP production (Du et al. 2017). In this study, we observed that FFAs significantly increased the levels of MDA, 8-OHdG, and significantly decreased the levels of ATP and mitochondrial membrane potential, suggesting that the mitochondrial function may be impaired. In contrast, the level of ATP and mitochondrial membrane potential significantly rebounded after YCLLT-MS treatment, implying that YCLLT may protect mitochondrial function.

Previous studies have shown that plant extracts or compounds can attenuate hepatic steatosis and ultimately ameliorate NAFLD by agonizing the AMPK pathway in hepatocytes (Chyau et al. 2020; Zhang et al. 2022). AMPK, a key regulator of bioenergetic metabolism, is inhibited by obesity, inflammation, and other factors associated with NAFLD. Activated AMPK regulates the activity of enzymes related to lipid metabolism and can be used to generate ATP by promoting lipolysis (Yan et al. 2022). Furthermore, activated AMPK can promote mitochondrial function by activating AMPK phosphorylation to accelerate energy metabolism (Xie et al. 2022). Studies have reported that AdipoR1 induces AMPK activation in the liver (Song et al. 2023). Meanwhile, changes in the expression of SIRT1 and PGC1α, as key downstream factors of AMPK, were tightly correlated with changes in AMPK phosphorylation, and PGC-1α, as a major factor in mitochondrial biosynthesis, regulated oxidative stress and inflammatory responses (Ji et al. 2023). Our study found that YCLLT-MS significantly activated the AdipoR1/AMPK/SIRT1 signaling pathway. To confirm the role of AdipoR1/AMPK/SIRT1 signaling pathway in the improvement of HepG2 cell steatosis by YCLLT, we silenced AdipoR1 to further explore the molecular mechanism of YCLLT, and the results showed that silencing of AdipoR1 inhibited the improvement of YCLLT in NAFLD cell model. In addition, in order to apply YCLLT treatment in the clinic for NAFLD patients in the future, experiments in mouse or rat models of NAFLD are needed to confirm the efficacy of YCLLT in attenuating lipid deposition, inhibiting inflammation, and ameliorating mitochondrial dysfunction. Also, the optimal dose range of YCLLT in animal models needs to be determined to ensure efficacy and safety. This will provide a basis for the future clinical application of YCLLT.

## Conclusions

This study proves that YCLLT was able to attenuate lipid deposition, inhibit inflammatory response, and ameliorate mitochondrial dysfunction in a NAFLD cell model, and the mechanism of its action may be related to the AdipoR1/AMPK/SIRT1 signaling pathway. However, there are some limitations to this study, in which we used only one cell line, and there is a need for validation in different cell models in the next studies. In addition, this experiment lacks in vivo experiments. In future studies, we will conduct more cell models (e.g., LO2 cells) and in vivo experiments to further validate the effect of YCLLT on NAFLD.

## Data Availability

The datasets used and/or analyzed during the current study are available from the corresponding author on reasonable request.
